# Papillary meningioma: a rare but distinct variant of malignant meningioma

**DOI:** 10.1186/1746-1596-2-3

**Published:** 2007-01-19

**Authors:** Singh Avninder, Sarvjot Vermani, Sharma Shruti, Karam Chand

**Affiliations:** 1 Institute of Pathology – ICMR, New Delhi, India; 2 Department of Neurosurgery, Safdarjang Hospital, New Delhi, India

## Abstract

**Background:**

Papillary meningiomas are rare meningeal tumors and are associated with aggressive clinical behavior as compared with other meningiomas. Because of their rare occurrence, they may pose a diagnostic dilemma to the unwary pathologist. We report a case of papillary meningioma in a 16-year-old boy.

**Case Presentation:**

A 16-year-old boy presented with complaints of headache, progressively diminishing vision and more recently generalized seizures. MRI revealed a large bifrontal meningioma which showed presence of a predominantly papillary pattern with areas of focal necrosis, frequent mitoses and bone invasion. He underwent radical excision of the tumor and is free from recurrence or metastasis at 15 months follow-up.

**Conclusion:**

Papillary meningiomas are rare but well recognized variants of meningioma. They need to be differentiated from other intracranial tumors with a papillary pattern. They are malignant, frequently show bone and parenchymatous invasion and have the potential for extracranial metastasis. Their timely recognition could prevent local and distant metastasis and the mortality or morbidity associated with it.

## Background

Meningiomas account for approximately 13–19% of all primary intracranial tumors and their prognosis is generally favorable [1]. Papillary meningioma (PM) is an aggressive histological variant of meningioma, which accounts for 1.0–2.5% of all meningiomas [2] and is defined by the presence of perivascular or pseudopapillary pattern either entirely or more commonly in combination with other common histological components of meningiomas. We report a case of PM in a 16-year-old boy and discuss its clinicopathologic findings.

## Case Presentation

**Figure 1:**
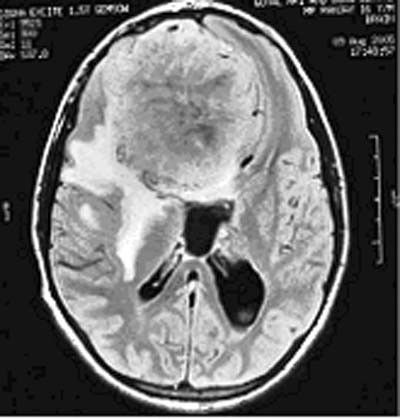
MRI scan showing a large bifrontal hyperintense mass with central necrosis.

A 16-year-old boy presented with a history of headache of 8 months duration, progressively decreasing vision for 3 months and more recently had an episode of generalized seizure 1 month back. Neurological examination did not reveal any focal neurological deficit except bilateral papilledema on fundus examination. MRI scan revealed a large 10 × 8 cm extra-axial hyperintense contrast enhancing mass lesion on the T1-weighted image, occupying the anterior cranial compartment [Fig 1]. The tumor was compressing the frontal horns of lateral ventricles along with anterior part of corpus callosum. It was also seen encroaching upon the superior saggital sinus. A bifrontal free bone craniotomy was performed and total excision of the tumor was achieved. Intraoperatively, the tumor was seen adhering to the frontal bone, a part of which was excised with the tumor.

**Figure 2:**
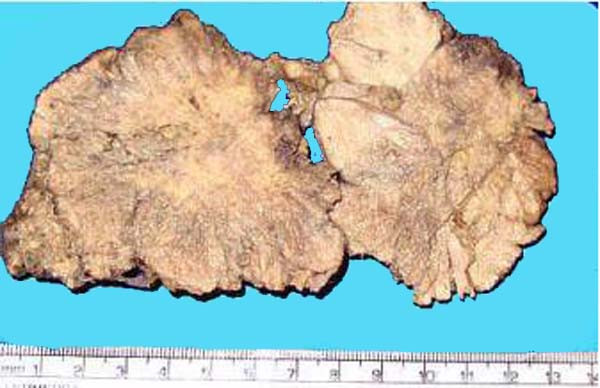
Cut-section of the tumor showing grayish-white irregular and friable surface.

**Figure 3:**
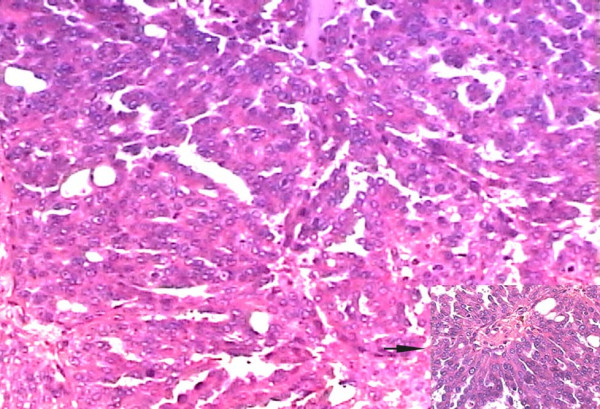
Photomicrograph of the tumor showing sheets of tumor cells arranged in papillary pattern and a papilla with fibrovascular core (inset) (H&E, ×200).

**Figure 4:**
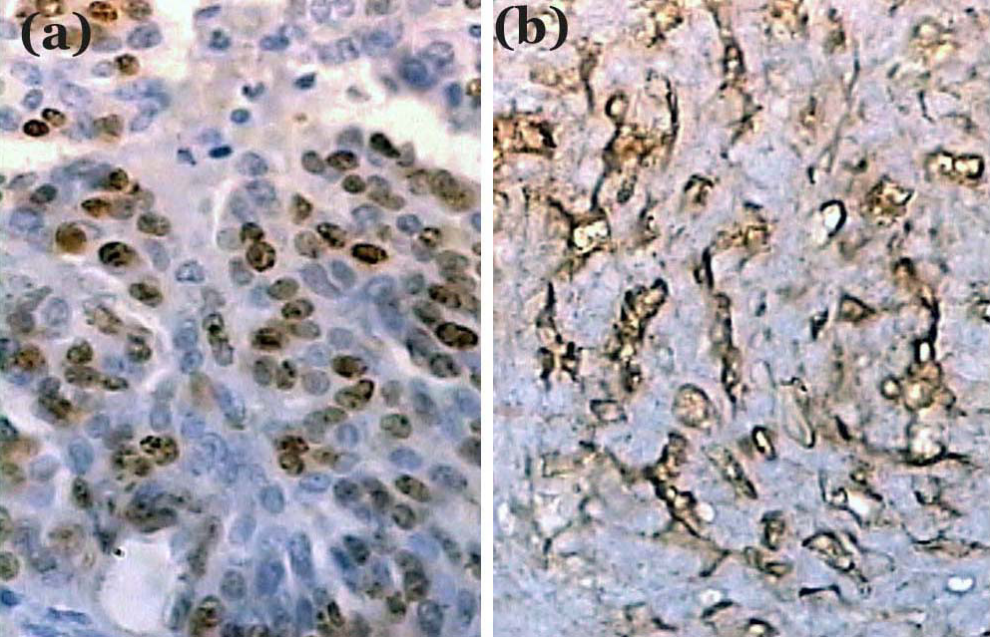
Tumor cells showing immunoreactivity to (a) MIB-1 and (b) EMA (×400).

Gross examination of the resected tumor showed a 10 × 8 × 2 cm soft tissue with its cut-section showing grayish-white, irregular surface with focal necrosis [Fig 2]. Histopathological examination revealed a tumor tissue composed of sheets of cells arranged in perivascular pseudopapillary pattern [Fig 3] along with few well formed papillae with central fibrovascular core [Fig 3, inset]. These papillary structures in some places were mixed with meningothelial sheets and whorls. The tumor cells in the papillary area displayed abundant eosinophilic cytoplasm, vesicular nuclei but frequent mitoses and foci of necrosis. MIB-1 labeling index was high with mean LI of 12% [Fig 4a]. Immunohistochemical positivity to epithelial membrane antigen (EMA) [Fig 4b] and vimentin was seen while Cytokeratin (CK), GFAP and S100 were non-reactive. A pathological diagnosis of Papillary meningioma, WHO grade 3 was given.

## Discussion

PM is a malignant variant of meningioma and was first described in 1938 by Cushing and Eisenhardt [3], in which they reported a papillary pattern in a meningioma showing intracerebral recurrence and pulmonary metastasis. The main diagnostic dilemma arises in the histological diagnosis of PM. The differential diagnoses include metastatic papillary adenocarcinoma, which is epithelial membrane antigen (EMA) and cytokeratin (CK) positive but vimentin negative; papillary ependymoma, which is glial fibrillary acidic protein (GFAP) positive; astroblastoma which is characterized by a typical palisaded pattern of astrocytic cells with broad and non-tapering strongly GFAP positive processes radiating towards the central blood vessels. Other tumors like choroids plexus papilloma may sometimes cause diagnostic confusion with a PM.

In one of the largest series of PM reported, Ludwin et al [4] described the clinicopathologic features of 17 cases and found that compared to other variants of meningiomas, PM were more frequent in children 8/17(47%). Mitoses were seen in 7/17(41%), local recurrences in 10/17(59%), brain invasion in 8/17(47%), and extracranial metastasis in 4/17(23.5%). This observation was supported by Brignolio and Favario [5] who studied 8 cases of PM and concluded that the presence of papillary pattern in meningioma corresponds with histological and clinical aggressiveness. Similarly, Radhakrishnan et al [6] reported 6 cases of PM all of which occurred in adults and most of them showed histological evidence of bone and brain invasion. In contrast, Stefanko and Mackay [7] described 6 cases of PM, none of which showed either local recurrence or distant metastasis. They had suggested that these papillary structures were no more than a secondary manifestation if tumor cell vasotropism and poor cohesion between cellular perivascular 'crowns'.

PM are frequently seen in the supratentorial compartment though rare locations like posterior fossa [8,9], jugular foramen [10] and occulomotor nerve [11] have been described. The presence of a tumor cyst is an exceptional finding in meningiomas, but it has been frequently reported in PM [12,13]. The standard treatment of PM is surgical resection and the effectiveness of adjuvant radiotherapy is yet to be established. Recurrences are frequent and lung is the most favored site of extracranial metastases [14,15]. The 5-year survival rate for PMs is about 40%, which is one-half for that of the other types of meningiomas [16]. In our 15 years of experience this was the first case of PM encountered and though the tumor was adherent to the bone at the time of extirpation, no recurrence or metastasis was seen after 15 months of follow-up. The patient had normal vision and is presently completely asymptomatic.

In meningiomas of higher grade, losses of chromosome 22q and 1p have been reported apart from mutations of the NF2 gene [17], but the genetic idiosyncrasies of PM are too scanty to be useful in making this ominous diagnosis.

## Conclusion

PM is exceptional variant of meningioma with high frequency in children, increased incidence of local recurrences and potential for distant metastasis. Their timely recognition may have important implications for management as well as prognostication in order to reduce the morbidity and mortality.

## Competing interests

The authors declare that they have no financial or non-financial competing interests and all the authors have read and approved the manuscript

## Authors' contributions

**SA **gave the histopathological diagnosis, took pictures and wrote the final draft of the manuscript. **SV **participated in grossing of the tumor, literature review, and modified the images and contributed suggestions for drafting the manuscript. **SS **participated in the histopathological diagnosis and **KC **was involved with the clinical management of the patient.
